# Supplementary material for: Single Nucleotide Polymorphisms in the Vitamin D Metabolic Pathway and Their Relationship with High Blood Pressure Risk
Source: Int J Mol Sci. 2023 Mar 22;24(6):5974. doi: 10.3390/ijms24065974 (PMC10057633; doi:10.3390/ijms24065974)
Supplement: Supplementary file 1 [file ijms-24-05974-s001.zip › Table S2.pdf]

Table S2. Linkage desequilibrium test results in the whole population.

Chr	Position (base pair)	SNP1	Gene	Chr	Position (base pair)	SNP2	Gene	R^2	D'
12	47844974	rs731236	*VDR*	12	47845054	rs7975232	*VDR*	0.52241	0.971043
12	47844974	rs731236	*VDR*	12	47846052	rs1544410	*VDR*	0.67492	0.898375
12	47845054	rs7975232	*VDR*	12	47846052	rs1544410	*VDR*	0.438847	0.838338
12	57764205	rs4646536	*CYP27B1*	12	57768115	rs3782130	*CYP27B1*	0.410515	0.853081
12	57764205	rs4646536	*CYP27B1*	12	57768302	rs10877012	*CYP27B1*	0.731762	0.905063
12	57764205	rs4646536	*CYP27B1*	12	57768956	rs703842	*CYP27B1*	0.747667	0.89186
12	57768115	rs3782130	*CYP27B1*	12	57768302	rs10877012	*CYP27B1*	0.386295	0.854472
12	57768115	rs3782130	*CYP27B1*	12	57768956	rs703842	*CYP27B1*	0.37292	0.836158
12	57768302	rs10877012	*CYP27B1*	12	57768956	rs703842	*CYP27B1*	0.758471	0.904393

Chr: Chromosome; SNP: Single Nucleotide Polymorphism.
