# Supplementary material for: Single Nucleotide Polymorphisms in the Vitamin D Metabolic Pathway and Their Relationship with High Blood Pressure Risk
Source: Int J Mol Sci. 2023 Mar 22;24(6):5974. doi: 10.3390/ijms24065974 (PMC10057633; doi:10.3390/ijms24065974)
Supplement: Supplementary file 1 [file ijms-24-05974-s001.zip › Table S3.pdf]

Table S3. Minor allele frequency of 13 SNPs in the whole population.

Chr	SNP	Gene	Minor Allele	Major Allele	MAF
4	rs7041	*GC*	T	G	0.4739
11	rs10741657	*CYP2R1*	A	G	0.369
12	rs731236	*VDR*	C	T	0.4021
12	rs7975232	*VDR*	C	A	0.4662
12	rs1544410	*VDR*	A	G	0.4169
12	rs2228570	*VDR*	T	C	0.358
12	rs11568820	*VDR*	A	G	0.2566
12	rs4646536	*CYP27B1*	G	A	0.2552
12	rs3782130	*CYP27B1*	C	G	0.305
12	rs10877012	*CYP27B1*	T	G	0.2468
12	rs703842	*CYP27B1*	C	T	0.2507
20	rs4809957	*CYP24A1*	G	A	0.2241
20	rs6068816	*CYP24A1*	T	C	0.1109

Chr: Chromosome; MAF: Minor Allele Frequency; SNP: Single Nucleotide Polymorphism.
