# Supplementary material for: Single Nucleotide Polymorphisms in the Vitamin D Metabolic Pathway and Their Relationship with High Blood Pressure Risk
Source: Int J Mol Sci. 2023 Mar 22;24(6):5974. doi: 10.3390/ijms24065974 (PMC10057633; doi:10.3390/ijms24065974)
Supplement: Supplementary file 1 [file ijms-24-05974-s001.zip › Table S5.pdf]

Table S5. Association of 13 SNPs with risk of HBP.

SNP	Gene	Minor Allele	Major Allele	Model	Cases	Controls	χ^2	p-value (χ^2 test)	p-value (FET)
rs7041	*GC*	T	G	Genotypic	44/122/76	114/252/120	4.841	0.08888	0.09042
				Additive	210/274	480/492	4.802	0.02843	0.02843
				Allelic	210/274	480/492	4.657	0.03093	0.03423
				Dominant	166/76	366/120	3.701	0.05438	0.06244
				Recessive	44/198	114/372	2.645	0.1039	0.106
rs10741657	*CYP2R1*	A	G	Genotypic	36/97/102	70/218/191	1.172	0.5565	0.5583
				Additive	169/301	358/600	0.256	0.6125	0.6125
				Allelic	169/301	358/600	0.270	0.6033	0.6407
				Dominant	133/102	288/191	0.811	0.3676	0.3743
				Recessive	36/199	70/409	0.062	0.8033	0.8231
rs731236	*VDR*	C	T	Genotypic	45/116/88	79/215/177	0.412	0.8136	0.8143
				Additive	206/292	373/569	0.406	0.5239	0.5239
				Allelic	206/292	373/569	0.424	0.515	0.5344
				Dominant	161/88	294/177	0.350	0.5536	0.5704
				Recessive	45/204	79/392	0.192	0.6605	0.6787
rs7975232	*VDR*	C	A	Genotypic	53/112/76	115/228/141	0.541	0.7628	0.7608
				Additive	218/264	458/510	0.531	0.4658	0.4658
				Allelic	218/264	458/510	0.562	0.4532	0.4679
				Dominant	165/76	343/141	0.443	0.5057	0.5469
				Recessive	53/188	115/369	0.282	0.5949	0.6408
rs1544410	*VDR*	A	G	Genotypic	49/106/88	94/220/177	0.139	0.9327	0.9256
				Additive	204/282	408/574	0.022	0.8808	0.8808
				Allelic	204/282	408/574	0.024	0.8758	0.9104
				Dominant	155/88	314/177	0.001	0.965	1
				Recessive	49/194	94/397	0.107	0.7426	0.7667
rs2228570	*VDR*	T	C	Genotypic	36/99/105	72/212/212	0.149	0.9281	0.9361
				Additive	171/309	356/636	0.008	0.9246	0.9246
				Allelic	171/309	356/636	0.009	0.9217	0.9538
				Dominant	135/105	284/212	0.067	0.7957	0.812
				Recessive	36/204	72/424	0.030	0.8619	0.9116
rs11568820	*VDR*	A	G	Genotypic	14/98/127	38/166/274	3.165	0.2055	0.2106
				Additive	126/352	242/714	0.176	0.6743	0.6743
				Allelic	126/352	242/714	0.182	0.669	0.7005
				Dominant	112/127	204/274	1.132	0.2874	0.3003
				Recessive	14/225	38/440	1.037	0.3086	0.3609
rs4646536	*CYP27B1*	G	A	Genotypic	14/88/139	40/172/268	1.482	0.4766	0.5108
				Additive	116/366	252/708	0.765	0.3816	0.3816
				Allelic	116/366	252/708	0.805	0.3696	0.4053
				Dominant	102/139	212/268	0.221	0.6378	0.6906
				Recessive	14/227	40/440	1.475	0.2245	0.2935
rs3782130	*CYP27B1*	C	G	Genotypic	33/85/114	48/144/217	1.250	0.5352	0.5317
				Additive	151/313	240/578	1.238	0.2658	0.2658
				Allelic	151/313	240/578	1.433	0.2313	0.2313
				Dominant	118/114	192/217	0.910	0.3401	0.366
				Recessive	33/199	48/361	0.830	0.3622	0.3874
rs10877012	*CYP27B1*	T	G	Genotypic	13/88/137	36/165/272	1.255	0.5338	0.5546
				Additive	114/362	237/709	0.198	0.6557	0.6557
				Allelic	114/362	237/709	0.207	0.6489	0.6958
				Dominant	101/137	201/272	0.002	0.9883	1
				Recessive	13/225	36/437	1.139	0.2858	0.3473
rs703842	*CYP27B1*	C	T	Genotypic	14/91/134	39/164/278	1.896	0.3875	0.4059
				Additive	119/359	242/720	0.011	0.9168	0.9168
				Allelic	119/359	242/720	0.011	0.9145	0.9486
				Dominant	105/134	203/278	0.195	0.6587	0.6895
				Recessive	14/225	39/442	1.186	0.2762	0.2939
rs4809957	*CYP24A1*	G	A	Genotypic	9/95/134	25/157/294	3.671	0.1595	0.1637
				Additive	113/363	207/745	0.738	0.3903	0.3903

				Allelic	113/363	207/745	0.727	0.3939	0.4193
				Dominant	104/134	182/294	1.972	0.1603	0.169
				Recessive	9/229	25/451	0.756	0.3844	0.4586
rs6068816	*CYP24A1*	T	C	Genotypic	4/45/192	11/84/381	NA	NA	0.8529
				Additive	53/429	106/846	0.005	0.9396	0.9396
				Allelic	53/429	106/846	0.006	0.9371	1
				Dominant	49/192	95/381	NA	NA	0.9216
				Recessive	4/237	11/465	NA	NA	0.7835

For Additive Model and Allelic Model, the counts shown in "Cases" and "Controls" are allele counts, not genotype counts, and for Genotypic Model, Dominant Model, and Recessive Model, the counts shown in "Cases" and "Controls" are genotype counts. Chr: Chromosome; FET: Fisher's exact test; HBP: High Blood Pressure; NA: Not Applicable; a: p-value for Bonferroni correction.
