# Supplementary material for: Single Nucleotide Polymorphisms in the Vitamin D Metabolic Pathway and Their Relationship with High Blood Pressure Risk
Source: Int J Mol Sci. 2023 Mar 22;24(6):5974. doi: 10.3390/ijms24065974 (PMC10057633; doi:10.3390/ijms24065974)
Supplement: Supplementary file 1 [file Table S1.pdf › Table S1.pdf]

Table S1. Hardy-Weinberg Equilibrium test results in the whole population, the case group, and the control group, respectively.

Chr	SNP	Gene	Sample	Minor Allele	Major Allele	Genotype counts	Observed heterozygosity	Expected heterozygosity	p-value
4	rs7041	*GC*	ALL	T	G	158/374/196	0.5137	0.4986	0.4573
			AFF	T	G	44/122/76	0.5041	0.4913	0.7934
			UNAFF	T	G	114/252/120	0.5185	0.4999	0.4678
11	rs10741657	*CYP2R1*	ALL	A	G	106/315/293	0.4412	0.4657	0.1717
			AFF	A	G	36/97/102	0.4128	0.4606	0.1194
			UNAFF	A	G	70/218/191	0.4551	0.4681	0.5584
12	rs731236	*VDR*	ALL	C	T	124/331/265	0.4597	0.4808	0.2452
			AFF	C	T	45/116/88	0.4659	0.4851	0.5164
			UNAFF	C	T	79/215/177	0.4565	0.4784	0.3355
12	rs7975232	*VDR*	ALL	C	A	168/340/217	0.469	0.4977	0.1178
			AFF	C	A	53/112/76	0.4647	0.4954	0.3628
			UNAFF	C	A	115/228/141	0.4711	0.4986	0.236
12	rs1544410	*VDR*	ALL	A	G	143/326/265	0.4441	0.4862	0.01877
			AFF	A	G	49/106/88	0.4362	0.4871	0.1139
			UNAFF	A	G	94/220/177	0.4481	0.4857	0.09423
12	rs2228570	*VDR*	ALL	T	C	108/311/317	0.4226	0.4597	0.03031
			AFF	T	C	36/99/105	0.4125	0.4587	0.1224
			UNAFF	T	C	72/212/212	0.4274	0.4602	0.1185
12	rs11568820	*VDR*	ALL	A	G	52/264/401	0.3682	0.3815	0.3779
			AFF	A	G	14/98/127	0.41	0.3882	0.5044
			UNAFF	A	G	38/166/274	0.3473	0.3781	0.08946
12	rs4646536	*CYP27B1*	ALL	G	A	54/260/407	0.3606	0.3801	0.1704
			AFF	G	A	14/88/139	0.3651	0.3655	1
			UNAFF	G	A	40/172/268	0.3583	0.3872	0.1001
12	rs3782130	*CYP27B1*	ALL	C	G	81/229/331	0.3573	0.4239	0.0861
			AFF	C	G	33/85/114	0.3664	0.4391	0.01613
			UNAFF	C	G	48/144/217	0.3521	0.4146	0.002769
12	rs10877012	*CYP27B1*	ALL	T	G	49/253/409	0.3558	0.3718	0.2669
			AFF	T	G	13/88/137	0.3697	0.3643	1
			UNAFF	T	G	36/165/272	0.3488	0.3755	0.1408
12	rs703842	*CYP27B1*	ALL	C	T	53/255/412	0.3542	0.3757	0.1363
			AFF	C	T	14/91/134	0.3808	0.374	0.8638
			UNAFF	C	T	39/164/278	0.341	0.3766	0.03971
20	rs4809957	*CYP24A1*	ALL	G	A	34/252/428	0.3529	0.3477	0.7474
			AFF	G	A	9/95/134	0.3992	0.3621	0.1513
			UNAFF	G	A	25/157/294	0.3298	0.3403	0.5012
20	rs6068816	*CYP24A1*	ALL	T	C	15/129/573	0.1799	0.1972	0.02306
			AFF	T	C	4/45/192	0.1867	0.1957	0.5031
			UNAFF	T	C	11/84/381	0.1765	0.1979	0.03246

Chr: Chromosome; ALL: All population; AFF: Case group; UNAFF: Control group; SNP: Single Nucleotide Polymorphisms
