# Supplementary material for: Single Nucleotide Polymorphisms in the Vitamin D Metabolic Pathway and Their Relationship with High Blood Pressure Risk
Source: Int J Mol Sci. 2023 Mar 22;24(6):5974. doi: 10.3390/ijms24065974 (PMC10057633; doi:10.3390/ijms24065974)
Supplement: Supplementary file 1 [file Table S4.pdf › Table S4.pdf]

Table S4. Haplotype frequency estimation for 6 SNPs located on Chromosome 12 in the whole population.

	rs1544410 (*VDR*)	rs7975232 (*VDR*)	rs731236 (*VDR*)	rs4646536 (*CYP27B1*)	rs703842 (*CYP27B1*)	rs10877012 (*CYP27B1*)	ALL	AFF	UNAFF	Cumulative frequency
1	G	C	T	A	T	G	0.3216	0.3437	0.317	0.3216
2	A	A	C	A	T	G	0.2349	0.2458	0.2287	0.5566
3	A	A	C	G	C	T	0.1134	0.1115	0.1132	0.6699
4	G	A	T	A	T	G	0.1011	0.0941	0.102	0.771
5	G	C	T	G	C	T	0.0738	0.071	0.0728	0.8448
6	A	C	T	A	T	G	0.0199	0.0165	0.0213	0.8647
7	G	A	T	G	C	T	0.0161	0.0181	0.0158	0.8809
8	G	A	C	A	T	G	0.0154	0.0174	0.0131	0.8963
9	A	A	T	A	T	G	0.0144	0.0184	0.0123	0.9107
10	G	C	T	G	T	G	0.0073	NA	0.0112	0.9179
11	G	A	C	G	C	T	0.0071	0.0173	0.0031	0.925
12	G	C	T	G	C	G	0.0059	0	0.0072	0.9309
13	A	C	C	A	T	G	0.0059	0.0046	0.0049	0.9368
14	G	C	T	A	C	T	0.0059	0	0.0072	0.9427
15	A	A	C	G	T	G	0.0057	0.0064	0.0052	0.9484
16	G	C	T	A	C	G	0.0053	0.0044	0.0056	0.9537
17	A	A	C	A	C	G	0.0045	0.0022	0.0054	0.9583
18	A	C	T	G	C	T	0.0045	0.0031	0.0054	0.9628
19	G	C	T	G	T	T	0.0043	NA	0.0066	0.967
20	A	A	C	G	C	G	0.0042	NA	0.0064	0.9712
21	G	C	T	A	T	T	0.004	0.0021	0.0048	0.9752
22	A	A	T	G	C	T	0.0039	0.0026	0.0043	0.9791
23	G	A	T	A	T	T	0.0035	NA	0.0059	0.9826
24	A	A	C	G	T	T	0.0034	NA	0.0049	0.9859
25	G	C	C	A	T	G	0.0022	NA	0.0032	0.9881
26	G	A	T	G	C	G	0.002	0.0041	0.0018	0.9901
27	G	A	T	A	C	T	0.0018	0.0021	0.0003	0.9919
28	A	A	C	A	T	T	0.0016	NA	0.0026	0.9935
29	A	C	T	A	C	G	0.001	NA	0	0.9945
30	G	A	T	G	T	T	9e-04	NA	0.0013	0.9954
31	G	A	C	A	C	T	8e-04	0.0022	NA	0.9962
32	G	C	C	A	C	G	8e-04	NA	0.0012	0.9969
33	G	C	C	G	T	T	7e-04	NA	0.0011	0.9976
34	A	A	T	G	T	G	7e-04	0.0021	NA	0.9983
35	A	A	T	G	C	G	6e-04	0.002	NA	0.9989
36	G	C	C	G	C	T	5e-04	NA	0.001	0.9994
37	G	A	T	A	C	G	5e-04	NA	0.002	0.9999
38	A	A	T	A	C	G	1e-04	0.0018	NA	1
39	A	C	C	G	C	T	0	0.0025	NA	1
40	A	C	C	A	C	G	0	NA	0.0014	1
41	A	A	C	A	C	T	0	0.0041	NA	1

ALL: All population; AFF: Case group; UNAFF: Control group; SNP: Single Nucleotide Polymorphism; NA: Not Applicable.
